# Opportunities and challenges in improving antimicrobial use during the era of telehealth expansion: A narrative review

**DOI:** 10.1017/ash.2021.191

**Published:** 2021-10-06

**Authors:** Hiroyuki Suzuki, Stephanie C. Shealy, Kyle Throneberry, Edward Stenehjem, Daniel Livorsi

**Affiliations:** 1 Center for Access & Delivery Research & Evaluation (CADRE), Iowa City Veterans’ Affairs Health Care System, Iowa City, Iowa; 2 Department of Internal Medicine, University of Iowa Carver College of Medicine, Iowa City, Iowa; 3 Intermountain Healthcare TeleHealth Services, Murray, Utah; 4 Department of Pharmacy, Intermountain Medical Center, Murray, Utah; 5 Division of Infectious Diseases and Clinical Epidemiology, Intermountain Healthcare, Salt Lake City, Utah

## Abstract

Efforts to improve antimicrobial prescribing are occurring within a changing healthcare landscape, which includes the expanded use of telehealth technology. The wider adoption of telehealth presents both challenges and opportunities for promoting antimicrobial stewardship. Telehealth provides 2 avenues for remote infectious disease (ID) specialists to improve inpatient antimicrobial prescribing: telehealth-supported antimicrobial stewardship and tele-ID consultations. Those 2 activities can work separately or synergistically. Studies on telehealth-supported antimicrobial stewardship have reported a reduction in inpatient antimicrobial prescribing, cost savings related to less antimicrobial use, a decrease in *Clostridioides difficile* infections, and improved antimicrobial susceptibility patterns for common organisms. Tele-ID consultation is associated with fewer hospital transfers, a shorter length of hospital stay, and decreased mortality. The implementation of these activities can be flexible depending on local needs and available resources, but several barriers may be encountered. Opportunities also exist to improve antimicrobial use in outpatient settings. Telehealth provides a more rapid mechanism for conducting outpatient ID consultations, and increasing use of telehealth for routine and urgent outpatient visits present new challenges for antimicrobial stewardship. In primary care, urgent care, and emergency care settings, unnecessary antimicrobial use for viral acute respiratory tract infections is common during telehealth encounters, as is the case for fact-to-face encounters. For some diagnoses, such as otitis media and pharyngitis, antimicrobials are further overprescribed via telehealth. Evidence is still lacking on the optimal stewardship strategies to improve antimicrobial prescribing during telehealth encounters in ambulatory care, but conventional outpatient stewardship strategies are likely transferable. Further work is warranted to fill this knowledge gap.

Antimicrobials are life-saving medications, and the use of these agents helps both treat and prevent infections. However, antimicrobial use is often unnecessary or suboptimal.^1,2^ Unnecessary antimicrobial use provides no benefit to the patient but exposes the patient to potential antimicrobial-related harms. Suboptimal antimicrobial use may also contribute to patient harm, particularly if antimicrobials are underdosed, if less effective agents are prescribed, or if overly broad-spectrum agents are used when more narrow-spectrum agents would suffice.

A major consequence of unnecessary and suboptimal antimicrobial use is antimicrobial resistance, which is an urgent public health threat. To address this problem, the need to improve antimicrobial prescribing is widely recognized. The Centers for Disease Control and Prevention (CDC), the World Health Organization (WHO), and multiple other organizations have prioritized the need to improve antimicrobial use.^3,4^ Since 2017, the Joint Commission has required that all accredited hospitals and nursing facilities have an antimicrobial stewardship program. In 2020, this requirement was also applied to all ambulatory healthcare organizations. In addition, the Centers for Medicaid and Medicare Services (CMS) has made the presence of an antimicrobial stewardship program a requirement for all participating hospitals and nursing facilities. Antimicrobial stewardship has 3 main goals: (1) optimizing clinical outcomes related to antimicrobial use, (2) minimizing toxicity and other adverse events related to antimicrobial use, and (3) limiting the emergence and spread of antimicrobial-resistant bacterial strains.^5^

Efforts to improve antimicrobial prescribing are occurring within a changing healthcare landscape, which includes the rapid expansion of telehealth services. Since it was first introduced in the 1990s, telehealth has been seen as a tool for increasing access and improving the quality of care for people living in resource-limited areas.^6^ However, the uptake of telehealth has been slow, partly due to inadequate reimbursements for telehealth visits.^7^ Telehealth use has dramatically increased during the coronavirus disease 2019 (COVID-19) pandemic in many outpatient settings because minimizing in-person care is a way to reduce viral transmission. Before the COVID-19 pandemic, telehealth visits accounted for ˜1% of all primary care physician (PCP) visits. In the second quarter of 2020 when the COVID-19 pandemic hit the United States, telehealth visits increased to 35% of all primary care visits and the total number of primary care visits decreased significantly.^8^

In this review, we discuss how telehealth intersects with both inpatient and outpatient antimicrobial prescribing. By sharing infectious disease (ID) expertise and supporting antimicrobial stewardship processes, telehealth can help to improve inpatient antimicrobial use. We review the evidence supporting the use of telehealth for this purpose, including identified barriers. We also discuss the benefits of outpatient tele-ID consultations and the challenges to improving outpatient antimicrobial use within telehealth-delivered ambulatory care, such as primary and urgent care.

## Definition of telehealth and telemedicine

The Healthcare and Public Health Sector Coordinating Council (HPH SCC) defines telehealth as “the use of telecommunications and information technology to provide access to health assessment, diagnosis, intervention, consultation, supervision, and information across distance.” Telemedicine is sometimes considered a subcategory of telehealth that refers to “the use of remote clinical services, encompassing diagnosis, treatment, and monitoring [of a patient].”^9^ However, some authors and organizations, such as the American Telemedicine Association (ATA), use telehealth and telemedicine as interchangeable terms.^10,11^ In this review, we use the term “telehealth” to describe the use of telecommunications and information technology to deliver care to a patient or to provide advice to a provider across a geographic distance. To minimize confusion, we do not distinguish between telemedicine and telehealth.

Synchronous telehealth refers to telehealth provided via a real-time encounter using encrypted audiovisual communication; it allows for a patient–physician interaction similar to that of face-to-face visits. Asynchronous telehealth, also called “store and forward” telehealth, refers to a mode of telehealth that lacks live video or audio interaction. Relevant clinical information such as chief complaints, pertinent patient history, laboratory results, and images are sent to a remote specialist for review. Using the collected information, the specialist formulates a recommendation and communicates with the patient and/or the physician. Both synchronous and asynchronous telehealth can be used for ID consultations and antimicrobial stewardship.

Although some studies define electronic consultation (e-consultation) as consultations using both synchronous and asynchronous telehealth, we define e-consultations as consultations using asynchronous telehealth similar to a recent systematic review.^12^ We use the term “tele-ID consultation” to capture ID consultation using synchronous telehealth, asynchronous telehealth, or both.

## Telehealth as a tool to improve inpatient antimicrobial prescribing

Telehealth can help share the expertise of ID specialists, including ID physicians, with resource-limited healthcare settings. Based on prior surveys, 41%–50% of US community hospitals lack an ID physician and 93% lack an ID-trained pharmacist.^13,14^ Many facilities, especially hospitals that are too small or too remote to justify having an on-site ID specialist, would benefit from using telehealth. The Center for Disease Control and Prevention (CDC) guidelines on the implementation of antimicrobial stewardship in small and critical-access hospitals state that the use of telehealth should be considered.^15^ The Infectious Diseases Society of America (IDSA) also supports appropriate and evidence-based use of telehealth to provide various kinds of ID services, including support for antimicrobial stewardship programs.^16^

Telehealth provides 2 avenues through which remote ID specialists can influence antimicrobial prescribing. First, remote ID specialists can support antimicrobial stewardship activities (ie, infectious diseases telehealth-supported antimicrobial stewardship, IDt ASP). Second, ID physicians can improve antimicrobial prescribing and associated clinical outcomes through direct tele-ID consultations. These 2 activities, which can be synergistic, work in different ways. Tele-ID consultation is initiated by a frontline physician’s consultation request. In contrast, IDt ASP can be a more proactive intervention because any patient on specific antimicrobials or with specific infectious diagnoses can be targeted without a frontline physician’s consultation request.

### Telehealth-supported antimicrobial stewardship

Table 1 shows a summary of studies on the implementation of IDt ASPs. IDt ASPs provide evidence-based antimicrobial stewardship expertise without an on-site ID specialist. The approach of IDt ASPs can be modified depending on available platforms and resources. Most IDt ASPs implement the strategy of prospective audit and feedback (PAF) to optimize antimicrobial therapy, which is endorsed as a cornerstone antimicrobial stewardship activity by the CDC and the Joint Commission.^17,18^ Prospective audit by ID telehealth (IDt) specialists can be achieved through remote or collaborative review with local caregivers and has been conducted at various frequencies (daily to biweekly). The IDt specialist must have EMR access for remote review, but if EMR access is lacking, the IDt specialist can collaboratively review the EMR with frontline caregivers. Communication of feedback and recommendations to frontline caregivers may be asynchronous (ie, EMR notes or e-mails)^19–25^ or synchronous (phone calls or teleconferences) with direct communication to local caregivers.^26–30^ All but 1 study involved a remote ID physician as a member of the IDt ASP.^19^ The level of engagement of local caregivers in the IDt ASP can also vary; some programs identifying local ASP champions such as pharmacists,^19,21,22,27–32^ physicians,^30^ or infection preventionists,^21,31^ and others operate remotely and independently. In addition to PAF, some IDt ASPs provide didactic and case-based sessions led by IDt specialists during synchronous teleconferences.^21,31^ Participants in the teleconferences reported a positive experience, including a better understanding of the rationale for recommendations provided in real-time communications. Participants also felt that teleconferences enabled discussions between frontline clinicians and the stewardship team, which was preferable to asynchronous forms of communication.^21^

**Table 1.** Summary of Studies for Telehealth-Supported Antimicrobial Stewardship Program

First Author, Year, Location	Settings	Study Design	Interventions	Member of Stewardship Team	Measured Outcomes	Comments
Wood, 2015, North Carolina, USA	Expansion of antimicrobial stewardship program to 6 community hospitals in the same healthcare system	Quasi-experimental study	Daily PAF for patients who is on controlled antimicrobials for >24 hours Chart review by ASP pharmacists at hub hospital with ID physician supervision Recommendations were conveyed by leaving a note in the EMR	ID physician at hub hospital ASP pharmacist at hub hospital	81%–95% of recommendations were accepted Decreased quinolone use in 3 of 6 hospitals Improved susceptibility of *Pseudomonas aeruginosa* to piperacillin/tazobactam in 1 hospital	All hospitals shared EMR
Knight, 2020, South Carolina, USA	Expansion of antimicrobial stewardship program to 6 community hospitals in the same healthcare system	Quasi-experimental study	Daily PAF for patients who is on targeted antimicrobials for ≥7 d Chart review by ASP pharmacists Recommendations were conveyed directly to provider (mainly via phone) or indirectly through on-site pharmacists ID physician was available for additional review or direct intervention per request	ID physician at hub hospital ASP pharmacist at hub hospital	91% of recommendations were accepted 4.6% reduction in antimicrobial use	All but 1 hospital shared EMR
Laible, 2019, South Dakota, USA	Expansion of antimicrobial stewardship program to 6 community hospitals in the same healthcare system	Quasi-experimental study	Daily PAF during 1-h teleconference between local pharmacists and remote ID physician Recommendations were conveyed from local pharmacists to local provider via in-person or verbally.	ID physicians at remote site Local pharmacists	Recommendation acceptance rate, 90%	
Stevenson, 2018, USA, Wilson, 2019, USA	Video-conference Antimicrobial Stewardship Team (VAST) for 2 rural VA hospitals	Quasi-experimental study	Weekly PAF during 1-h teleconference involving multidisciplinary team Recommendations were recorded in the EMR ID e-consultation available outside teleconference	ID physicians at remote site Multidisciplinary team (pharmacist champion at 1 site, and infection preventionist champion at another site)	Recommendation acceptance rates, 65% and 73% Decreased antimicrobial days of therapy Decreased mean antibiotic spectrum index (ASI)	VAST – combined tele-stewardship and tele-consultation Hospitals have both acute-care beds and long-term acute-care beds
Yam, 2012, Washington, USA	Development of pharmacist-red antimicrobial stewardship program in a community hospital with support of remote ID physician	Quasi-experimental study	Weekly PAF during 30-min teleconference between local pharmacists and remote ID physician Recommendations conveyed via written communication form created by local pharmacists ID e-consultation per request	ID physicians at remote site Local pharmacists	Decrease in antimicrobial cost by 28% Nosocomial CDI decreased from 5.5 to 1.6 per 10,000 patient days	
Shively, 2020, Pennsylvania, USA	Expansion of antimicrobial stewardship program to 2 community hospitals in a different healthcare system	Quasi-experimental study	2–3 times/week PAF during 1-h teleconference between local pharmacists and remote ID physician Recommendations conveyed via in-person or telephone communication from local pharmacists	ID physicians at remote site Local pharmacists	Recommendation acceptance rate, 89% DOT for broad-spectrum antimicrobials decreased by 24% Annual cost savings on antimicrobial expenditures, USD 142K ID consultation increased by 40%	Remote ID physician had access to patient charts
Stenehjem, 2018, Utah and Idaho, USA	Implementation of telehealth-supported antimicrobial stewardship strategies among 15 community hospitals	Cluster-randomized trial	Strategy 1: basic AS education tools + ID hotline Strategy 2: 1+ advanced AS education, limited audit and feedback by local pharmacist and local antimicrobial restriction Strategy 3: 1+ advanced AS education, expanded audit and feedback, ID-controlled antimicrobial restriction, and ID review of designated cultures	ID physicians and ID pharmacists at remote site Local pharmacists	DOT for total antimicrobials and broad-spectrum antimicrobials significantly decreased in hospitals with strategy 3 No significant differences in mortality, 30-d readmission, and hospital length of stay	
Vento, 2021, Utah and Idaho, USA	Same as Stenehjem’s study, expanding to 16 community hospitals	Retrospective cohort study	Daily PAF by remote IDt pharmacists triggered by Vigilanz alert Recommendations were conveyed to local pharmacists via phone call	ID physicians and ID pharmacists at remote site Local physicians and pharmacists	Recommendation acceptance rate, 88% DOT for fluoroquinolone (147 to 82 per 1,000 days present), meropenem (13 to 9 per 1,000 days present), and vancomycin (75 to 53 per 1,000 days present) decreased 8 quality improvement projects conducted	
Beaulac, 2016, Massachusetts, USA	Expansion of antimicrobial stewardship to a long-term acute-care hospital	Quasi-experimental study	Daily PAF by remote ASP team for patients on targeted antimicrobials for ≥7 d Recommendations were conveyed through daily emails	ID physicians and ASP pharmacists at remote site	Significantly decreased antimicrobial use by 6.58 DDD/1,000 patient days Hospital-acquired CDI decreased by 43%	Remote ID physician had full access to local EMR
Dos Santos, 2012 and 2019, Brazil	Development of antimicrobial stewardship program in a remote community hospital with support of remote ID physician	Quasi-experimental study	Immediate PAF by remote ID physicians after prescription of antimicrobials Recommendations were conveyed via web platform, email or text message	ID physicians at remote site	Decreased usage of several classes of antimicrobials (fluoroquinolones, carbapenems, etc) Increased appropriateness of antimicrobials (51 to 81%) Decreased rate of carbapenem-resistant *Acinetobacter* spp	Remote ID physician assessed appropriateness through web platform
Howell, 2019, Texas, USA	Expansion of pharmacy-led antimicrobial stewardship program to a community hospital in the same healthcare system	Quasi-experimental study	Daily PAF by remote ASP pharmacists triggered by Theradoc alert Recommendations conveyed to local pharmacists via Theradoc Local pharmacists communicate with local providers as appropriate	ASP pharmacists at remote site Local pharmacists	Recommendation acceptance rate, 17% 48% of patients for possible intervention had already discharged Average USD 280 cost savings per patient in pharmacy charges	Hospitals shared EMR and Theradoc
Ceradini, 2017, Italy	Development of antimicrobial stewardship program in pediatric cardiac hospital with support of remote ID physician	Quasi-experimental study	Biweekly PAF during teleconference involving multidisciplinary team	ID physician and microbiology specialist at remote site Cardiologists, anesthesiologists and surgeon at local hospital	Decreased rate of nosocomial infection (9.5 to 6.5 per 1,000 person days) Decreased isolation of multidrug resistant organism (104 to 79 per 1,000 person days) Antimicrobial-related cost reduction (43 to 27 euros per admission)	

Note. PAF, prospective audit and feedback; ID, infectious diseases; EMR, electronic medical record; ASP, antimicrobial stewardship program; VA: Veterans’ Affairs; CDI,*Clostridioides difficile* infection; DOT, days of therapy; AS, antimicrobial stewardship; IDt, infectious diseases telehealth; DDD, defined daily doses.

Studies on IDt ASPs have reported improved outcomes such as decreased inpatient antimicrobial use,^20,23–26,28–31^ decreased cost related to inpatient antimicrobials,^19,22,32^ decreased *Clostridioides difficile* infections,^22,23^ and improved antimicrobial susceptibility patterns of common organisms.^20,24,25,32^ One study also reported a 40% increase in ID consultations after the implementation of IDt ASP.^28^

Several unique challenges have been identified in previous studies. First, the timeliness of remote ID specialists reviewing patients on active antimicrobials may be a barrier. Opportunities for improving antimicrobial prescribing can be missed if PAF is not performed frequently, especially when patient turnover is rapid. In one study, only 17% of recommendations were accepted, and 48% of stewardship recommendations were for patients who had already been discharged.^19^ In this study, communication was delayed because a remote stewardship pharmacist reviewed cases at the end of each weekday and passed on recommendations to a local pharmacist, who might not have acted on the feedback until the following workday. Another barrier to IDt ASP could be technical difficulties, such as audio interference, especially at the beginning of implementation.^21^ Other barriers, not limited to telehealth, were limitations in resources and interference with other clinical duties.^27^

Overall, IDt ASPs appear to achieve outcomes similar to those of traditional antimicrobial stewardship programs. Further studies are needed to characterize the optimal model for IDt ASP activities and communications.

### Telehealth for remote inpatient ID consultation

A summary of studies on tele-ID consultation is provided in Table 2. Telehealth has been used as an alternative way to consult with ID physicians for both hospitalized patients and patients in ambulatory care. For inpatient settings, tele-ID consultation has been used as a way to provide ID expertise for hospitals without an on-site ID physician.^30,33–35^ IDt specialists can provide advice on the initiation of empiric antibiotics, therapy modifications based on culture results and patients’ clinical response, or transfer to higher-level care when needed. Additionally, tele-ID inpatient consultations provide an opportunity for an ID physician to establish a therapeutic relationship with a patient, to arrange in-person or direct-to-consumer video follow-up visits and/or to arrange outpatient parenteral antimicrobial therapy.

**Table 2.** Summary of Studies for Tele-ID Consultation

First Author, Year, Location	Settings and Modality of Telehealth	Study Design	Reasons for Consultation	Findings	Comments
Tande, 2020, Minnesota, USA	Inpatient asynchronous e-consult to 2 community hospitals within the same healthcare system	Case–control study	Not stated	Decreased 30-d mortality in patients with e-consultation (adjusted odds ratio, 0.3; 95% CI, 0.2–0.7) No significant difference in readmission and hospital transfer Longer hospital stay in patients with e-consult (OR, 1.3; 95% CI, 1.2–1.5) 95% of referring providers were very satisfied with e-consultation	Propensity-score matched using patient age, gender, race, and weighted Charlson Comorbidity Index
Canterino, 2021, Connecticut, USA	Inpatient tele-ID consultation (either synchronous or asynchronous) within a single hospital (with 2 campuses)	Qualitative study	Not stated	80% agreed that electronic consult provided good clinical care Quality: the same or better in 67% Timeliness: the same or better in 99% Communication: the same or better in 80% ID consultants more likely felt overall quality was worse than traditional ID consult ID consultants more likely felt there were specific situations where face-to-face consultation was necessary	Survey conducted via e-mail Response rate: 23.6% Conducted during COVID-19 pandemic
Assimacopoulos, 2008, South Dakota, USA	Inpatient synchronous tele-ID consultation to nine community hospitals within the same healthcare system	Retrospective cohort study	Neutropenic fever Bacterial pneumonia Bacterial wound infection	Compared to in-person ID consultation at a hub hospital, shorter intravenous antimicrobial treatment days (13.4 vs 6.9 d) and shorter hospital length of stay (10.7 vs 6.5 d) No difference in % of survival and transfer	
Monkowski, 2020, Pennsylvania, USA	Inpatient synchronous tele-ID consultation to a community hospital within the same healthcare system	Retrospective cohort study	Not stated	Hospital transfer decreased from 100% to 8% No significant difference in readmission and ICU admission Decrease in total length of hospital stay from 14 to 9 days Antimicrobial-related cost reduction from 14.5 to 11.3 USD per day	
Strymish, 2017, Massachusetts, USA	Outpatient and inpatient asynchronous e-consultation at a VA healthcare system	Quasi-experimental study	Antimicrobial use for bacterial infection, 33% Latent tuberculosis, 14%	Time to completion for e-consults averaged 0.6 days compared to 16.5 days for face-to-face visits Volume of consultation increased from 480 (285 e-consults and 195 face-to-face) compared to pre-implementation of e-consult (193 face-to-face) without decreasing the volume of face-to-face visits	
Murthy, 2017, Eastern Ontario, Canada	Outpatient asynchronous e-consultation for primary care providers in the same healthcare network	Prospective cohort study	Tuberculosis, 14% Lyme disease, 14% Parasite infections, 13% Vaccination, 10%	Mean length of time needed to the response was 8 h 63% got response within 24 h 89% rated 4 or greater in the 5-point Likert scale about value of e-consultation (higher is better) 55% of PCP felt they got additional information impacting on patient care 40% of PCP felt their action was confirmed by ID Only 2% of PCP felt e-consultation was not very useful	
Mashru, 2017, Northwestern Ontario, Canada	Outpatient direct-to-consumer synchronous telehealth	Prospective cohort study	Musculoskeletal infection, 26% Skin and soft-tissue infection, 23%	Overall very high patient satisfaction, >98%	82% of the covered population was a First Nations population
Wood, 2020, Washington, USA	Outpatient asynchronous e-consultation for primary care providers in the same healthcare network	Retrospective cohort study	Interpretation of positive culture, PCR or serology for specific organism, 10% Syphilis, 10% Latent tuberculosis, 9%	Mean days to response, 0.7 d Decreased number of internal calls for curbside ID consultation (mean, 5.8–2.9 per month; *P* = .022) No significant difference in number of face-to-face ID consultation and time to in-person ID consultation	
Gonzalez, 2021, Ohio, USA	Outpatient asynchronous e-consultation for pediatric primary care providers in the same healthcare system	Cross-sectional study	Vaccination, 25% Exposures, 22%	RVUs for 197 e-consultation were equivalent in effort to 70 level-4 initial outpatient consults	
Vento, 2021, Utah and Idaho, USA	Phone advice, asynchronous e-consultation and inpatient synchronous tele-consultation to 16 community hospitals in the same healthcare system	Retrospective cohort study	Bloodstream infection, 39% Musculoskeletal infection, 20% Skin and soft-tissue infection, 10%	35% were phone advice only, 30% were e-consultation and 35% were teleconsultation >90% of patients and referring provider felt improved care, and telehealth was necessary and easy to use	

Note. ID, infectious diseases; CI, confidence interval; ICU, intensive care unit; VA, Veterans’ Affairs; PCP, primary care physician; PCR, polymerase chain reaction; RVU: relative value unit.

Inpatient tele-ID consultation has been associated with fewer hospital transfers,^33^ shorter hospital length of stay^33,34^ and decreased 30-day mortality.^35^ Generally, physicians have been very satisfied with inpatient tele-ID consultation.^30,35,36^ However, in one study, in which inpatient tele-ID consultation was used as an alternative to in-person ID consultation during the COVID-19 pandemic, ID consultants felt that the overall quality of tele-ID consultation was worse than traditional in-person ID consultation and that there are specific situations in which in-person consultation is necessary.^36^

Access to patient EMRs, laboratory results, and imaging studies is necessary for both synchronous and asynchronous tele-ID consultation. In fact, all of the studies cited here were conducted in the same healthcare system in which remote ID specialists had full access to the EMR of the local hospitals.^30,33–36^ For inpatient synchronous direct-to-consumer tele-ID consultation, a nurse or a local provider is often in the patient’s room, and additional equipment may be needed, such as in-room examination cameras or electronic stethoscopes.^30,34^ Some potential hurdles for implementing tele-ID consultation include medical licensure, medical liability insurance, and reimbursement across state lines.^37^ Because tele-ID consultations are often combined with IDt ASP, offering an annual subscription to both telehealth service may be a good model.^30^

### Integration of tele-ID consultation and telehealth-supported antimicrobial stewardship

Hospitals can simultaneously implement tele-ID consultation and IDt ASP. In fact, combined IDt ASP and e-consultation, called a videoconference antimicrobial stewardship team (VAST), was successfully implemented at 2 medical centers in the Veterans’ Affairs health system.^21,31^ In the VAST program, a remote ID physician provided input on selected ID cases during weekly video conferences with local hospital staff members.^31^ Similarly, Vento et al^30^ described integrated ID telehealth services, which included tele-ID consultation and IDt ASP.

Previous studies have suggested that antimicrobial stewardship activities stimulate formal ID consultation rather than replace it.^38,39^ Likewise, both telehealth-supported antimicrobial stewardship and e-consultation have increased the number of total ID consultations without decreasing the number of in-person ID consultations.^28,29,40^

Notably, for complicated cases, in-person ID consultation has distinct advantages to remote, telehealth reviews. However, a recent retrospective study reported that tele-ID consultation was as effective as in-person ID consultation for patients with *Staphylococcus aureus* bacteremia when a care bundle for *S. aureus* bacteremia had already been implemented by the local antimicrobial stewardship team.^41^ This study highlights the importance of collaboration between tele-ID consultation and telehealth-supported antimicrobial stewardship activities.

## Telehealth and outpatient antimicrobial use

### Telehealth to access ID expertise for outpatients

In outpatient settings, e-consultations can be used to more rapidly access ID expertise (Table 2).^30,40,42–44^ Similar to inpatient tele-ID consultation, referring physicians expressed satisfaction with e-consultations either by learning additional information that affected patient care or by receiving validation of their management decisions.^44^ Interestingly, a large number of outpatient e-consultations were related to questions that had previously been posed to ID consultants via curbside consultations; these informal discussions did not involve the consultant seeing the patient or reviewing the EMR. Questions centered around the interpretation of laboratory results, antimicrobial recommendation for positive microbiology results, vaccinations, or particular exposures. In fact, one study reported a decreased number of curbside ID consultations after implementation of outpatient e-consultations.^43^ Therefore, outpatient e-consultation can be regarded as a preferable alternative to telephone calls, which can increase access to ID expertise rather than replace in-person ID consultation.

One study implemented direct-to-consumer synchronous telehealth as a way to expand ID expertise to outpatients in a remote region where most of the population was part of First Nations communities.^45^ Over the course of 1 year, this project provided both direct patient care and held case conferences with providers. Patient satisfaction was high.

### Antimicrobial stewardship opportunities for telehealth-delivered primary, urgent, and emergency department care

Telehealth is increasingly used as a tool for providing care in outpatient settings, including primary care, urgent care, and emergency departments. Telehealth visits in ambulatory care offer opportunities for antimicrobial stewardship. Some challenges to improving antimicrobial use in these telehealth visits are new, and other challenges are similar to those encountered in more traditional outpatient settings.

### Factors affecting antimicrobial prescribing in outpatient telehealth visits

The decision to prescribe antimicrobials in an outpatient setting is a complex issue influenced by many external and internal factors. Based on prior studies of face-to-face encounters in ambulatory care, physician factors that contribute to antimicrobial overuse include knowledge deficits or lack of familiarity with treatment guidelines, diagnostic uncertainty, and a desire to ensure patient satisfaction.^46^ In addition to physician factors, antimicrobial prescribing can be affected by patient factors. For example, patient pressure or expectation to receive antimicrobials can lead to antimicrobial overprescription. Other patient factors include patient comorbidities (immunosuppression, etc), socioeconomic status, and communication barriers. External factors, such as organizational pressure for financial incentives, further complicate the decision making for antimicrobial prescribing. Physicians may elect to prescribe antimicrobials to see more patients rather than taking more time to explain why antimicrobials are not indicated.

Most of the aforementioned factors, which were identified from research on face-to-face encounters, may hold true in the setting of telehealth, but some factors may be stronger and others may be weaker. Gomez et al^47^ conducted a qualitative study to assess physician perspectives on telehealth. Primary care physicians uniformly stated that their inability to perform a physical examination is a major disadvantage to telehealth, and some also thought that this could lead to antimicrobial overprescribing. In the same study, several physicians stated that they felt more comfortable refusing patient requests for unnecessary antimicrobials during telehealth visits compared to face-to-face visits.

### Previous studies about frontline providers’ antimicrobial prescribing via telehealth

Several studies have compared frontline providers’ antimicrobial prescribing via telehealth and face-to-face visits with mixed results. These studies varied in the type of telehealth, type of face-to-face visits, and infectious diagnoses for which antimicrobials were prescribed; settings included primary care, urgent care, emergency care, and retail clinics. A systematic review and meta-analysis of studies that compared antimicrobial prescribing via telehealth and face-to-face encounters for common outpatient infections found that antimicrobials were more frequently prescribed via telehealth compared to face-to-face visits for patients with otitis media (pooled odds ratio [OR], 1.26; 95% confidence interval [CI], 1.04–1.52) and pharyngitis (pooled OR, 1.16; 95% CI, 1.01–1.33).^48^ No significant differences were observed between the 2 modes of delivery for sinusitis and upper respiratory infection (URI). Across both modes of care delivery, antimicrobials were prescribed for 30%–40% of patients with URI, an infection in which antimicrobials are almost never indicated, and antimicrobials were prescribed for 60%–70% of patients with sinusitis, otitis media, and pharyngitis—3 infections that are frequently viral. Due to significant heterogeneity across the included studies, these results need further validation. Nevertheless, these findings suggest that antimicrobials are commonly overprescribed in outpatient settings and might be further overprescribed via telehealth for diagnoses in which physical examination is a necessary part of evaluation, such as otitis media and pharyngitis. It is possible that increased diagnostic uncertainty due to the lack of physical examination resulted in more antimicrobial use, but there is a need for more research to understand how antimicrobial decision making in telehealth differs from decision making in face-to-face encounters.

### Strategies to improve outpatient providers’ antimicrobial prescribing for telehealth encounters

Conventional outpatient antimicrobial stewardship strategies might also be effective in telehealth settings that replace traditional primary, urgent care, or emergency room care. All of the CDC core elements for outpatient antibiotic stewardship are applicable to the telehealth setting.^49^ In addition, many policies or practices, such as delayed prescribing or requiring written justification, can be applied in the EMR and will work for both telehealth and face-to-face visit settings.

However, research on implementing stewardship strategies within the context of telehealth encounters is limited. In a study across a primary care network in western Michigan, a computerized decision support system (CDSS) was leveraged as a stewardship strategy. This CDSS guided physicians to the correct diagnosis and treatment using drop-down menus of guideline-based treatment options. The authors observed significantly more guideline-concordant diagnoses (69.1% vs 45.7%) and less antimicrobial prescribing (68.6% vs 94.3%) for patients with sinusitis in telehealth compared to face-to-face visits.^50^ Similarly, patients with urinary tract infection seen via telehealth were more likely to receive firstline antibiotic agents (74.9% vs 59.4%) and a guideline-concordant duration of therapy (100% vs 53.1%) compared to those seen via face-to-face visits.^51^

Pedrotti et al^52^ described antibiotic-prescribing practices during synchronous direct-to-consumer telehealth for outpatient visits in Brazil. All telehealth doctors were general practitioners; they were trained on antibiotic stewardship protocols, monitored by senior supervisors, and provided bimonthly feedback on their protocol adherence. Within this framework, rates of antimicrobial prescribing were low for conditions that never or frequently do not require antibiotics, such as URIs (2.5% received antimicrobials), pharyngo-tonsillitis (35% received antimicrobials), acute sinusitis (51.8% received antimicrobials), and acute diarrhea (1.6% received antimicrobials).

Although most outpatient antimicrobial stewardship interventions may work for antimicrobial prescribing via telehealth, the adoption of antimicrobial stewardship in outpatient settings, in general, continues to lag.^53^ Continued efforts to expand the reach of outpatient antimicrobial stewardship activities will likely lead to improved antimicrobial prescribing via telehealth. In addition, it is important to acknowledge that patients with some diagnoses that require physical examination may be at risk for antimicrobial overprescribing when seen via telehealth. Additional measures should be undertaken to avoid this risk. One possible solution is to deploy tools that enable remote physical examination, such as a remote stethoscopes or remote otoscopes. Another solution would be to develop guidelines for schedulers and triage officers to guide decisions about types of visits that are appropriate for telehealth versus face-to-face visits.^47^

In this narrative review, we have discussed how telehealth can provide access to remote ID specialists and, in turn, can be an effective tool for improving both antimicrobial use and clinical outcomes in patients treated with antimicrobials. There is tremendous potential to expand the reach of ID specialist and antimicrobial stewardship expertise through telehealth, but additional research is needed to define optimal strategies for implementing this technology and to adapt it to different local settings.

Routine medical care delivered via telehealth also presents new challenges and opportunities for efforts to promote antimicrobial stewardship. It is unclear whether existing stewardship strategies may transfer easily from the face-to-face to the telehealth setting. The increased adoption of telehealth during the COVID-19 pandemic will probably encourage high levels of telehealth use even after the pandemic has ended.^54^ Given the continued use of telehealth services and the urgent public health threat of antimicrobial resistance, further work is needed to evaluate the effectiveness and optimal implementation of stewardship strategies within these types of encounters.

## References

[1] Magill SS , O’Leary E , Ray SM , et al. Assessment of the appropriateness of antimicrobial use in US hospitals. JAMA Netw Open 2021;4:e212007. 33734417 10.1001/jamanetworkopen.2021.2007 PMC7974639

[2] Fleming-Dutra KE , Hersh AL , Shapiro DJ , et al. Prevalence of inappropriate antibiotic prescriptions among US ambulatory care visits, 2010–2011. JAMA 2016;315:1864–1873. 27139059 10.1001/jama.2016.4151

[3] Antibiotic resistance threats in the United States, 2019. Centers for Disease Control and Prevention website. https://www.cdc.gov/drugresistance/pdf/threats-report/2019-ar-threats-report-508.pdf. Accessed June 24, 2021.

[4] Global action plan on antimicrobial resistance. World Health Organization website. https://www.who.int/publications/i/item/9789241509763. Accessed June 24, 2021.

[5] Policy statement on antimicrobial stewardship by the Society for Healthcare Epidemiology of America (SHEA), the Infectious Diseases Society of America (IDSA), and the Pediatric Infectious Diseases Society (PIDS). *Infect Control Hosp Epidemiol* 2012;33:322–327. 10.1086/665010 22418625

[6] Bashshur RL. Telemedicine effects: cost, quality, and access. J Med Syst 1995;19:81–91. 7602255 10.1007/BF02257059

[7] Mehrotra A , Jena AB , Busch AB , Souza J , Uscher-Pines L , Landon BE. Utilization of telemedicine among rural medicare beneficiaries. JAMA 2016;315:2015–2016. 27163991 10.1001/jama.2016.2186 PMC4943212

[8] Alexander GC , Tajanlangit M , Heyward J , Mansour O , Qato DM , Stafford RS. Use and content of primary care office-based vs telemedicine care visits during the COVID-19 pandemic in the US. JAMA Netw Open 2020;3:e2021476. 33006622 10.1001/jamanetworkopen.2020.21476 PMC7532385

[9] Health Industry Cybersecurity–Securing Telehealth and Telemedicine (HIC-STAT). Health Sector Council website. https://healthsectorcouncil.org/securingtelehealth/. Accessed July 26, 2021.

[10] Hoffman LC. Shedding light on telemedicine and online prescribing: the need to balance access to health care and quality of care. Am J Law Med 2020;46:237–251. 32659190 10.1177/0098858820933497

[11] Tuckson RV , Edmunds M , Hodgkins ML. Telehealth . N Engl J Med 2017;377:1585–1592. 29045204 10.1056/NEJMsr1503323

[12] Vimalananda VG , Orlander JD , Afable MK , et al. Electronic consultations (e-consults) and their outcomes: a systematic review. J Am Med Inform Assoc 2020;27:471–479. 31621847 10.1093/jamia/ocz185 PMC7647247

[13] Septimus EJ , Owens RC , Jr. Need and potential of antimicrobial stewardship in community hospitals. Clin Infect Dis 2011;53:S8–S14. 21795728 10.1093/cid/cir363

[14] Reese SM , Gilmartin H , Rich KL , Price CS. Infection prevention needs assessment in Colorado hospitals: rural and urban settings. Am J Infect Control 2014;42:597–601. 24837109 10.1016/j.ajic.2014.03.004

[15] Implementation of antibiotic stewardship core elements at small and critical access hospitals. Centers for Disease Control and Prevention website. https://www.cdc.gov/antibiotic-use/healthcare/pdfs/core-elements-small-critical.pdf. Accessed May 18, 2021.

[16] Siddiqui J , Herchline T , Kahlon S , et al. Infectious Diseases Society of America position statement on telehealth and telemedicine as applied to the practice of infectious diseases. Clin Infect Dis 2017;64:237–242. 28096274 10.1093/cid/ciw773

[17] The core elements of hospital antibiotic stewardship programs: 2019. Centers for Disease Control and Prevention website. https://www.cdc.gov/antibiotic-use/healthcare/pdfs/hospital-core-elements-H.pdf. Accessed August 3, 2021.

[18] New antimicrobial stewardship standard. The Joint Commission website. https://www.jointcommission.org/-/media/enterprise/tjc/imported-resource-assets/documents/new_antimicrobial_stewardship_standardpdf.pdf?db=web&hash=69307456CCE435B134854392C7FA7D76. Accessed May 19, 2021.

[19] Howell CK , Jacob J , Mok S. Remote antimicrobial stewardship: a solution for meeting the joint commission stewardship standard? Hosp Pharm 2019;54:51–56. 30718935 10.1177/0018578718769240 PMC6333956

[20] Wood ZH , Nicolsen NC , Allen N , Cook PP. Remote antimicrobial stewardship in community hospitals. Antibiotics (Basel) 2015;4:605–616. 27025642 10.3390/antibiotics4040605 PMC4790314

[21] Stevenson LD , Banks RE , Stryczek KC , et al. A pilot study using telehealth to implement antimicrobial stewardship at two rural Veterans’ Affairs medical centers. Infect Control Hosp Epidemiol 2018;39:1163–1169. 30185238 10.1017/ice.2018.197

[22] Yam P , Fales D , Jemison J , Gillum M , Bernstein M. Implementation of an antimicrobial stewardship program in a rural hospital. Am J Health Syst Pharm 2012;69:1142–1148. 22722593 10.2146/ajhp110512

[23] Beaulac K , Corcione S , Epstein L , Davidson LE , Doron S. Antimicrobial stewardship in a long-term acute-care hospital using offsite electronic medical record audit. Infect Control Hosp Epidemiol 2016;37:433–439. 26752662 10.1017/ice.2015.319

[24] Dos Santos RP , Dalmora CH , Lukasewicz SA , et al. Antimicrobial stewardship through telemedicine and its impact on multidrug resistance. J Telemed Telecare 2019;25:294–300. 29720043 10.1177/1357633X18767702

[25] dos Santos RP , Deutschendorf C , Carvalho OF , Timm R , Sparenberg A. Antimicrobial stewardship through telemedicine in a community hospital in southern Brazil. J Telemed Telecare 2013;19:1–4. 23390213 10.1177/1357633X12473901

[26] Knight J , Michal J , Milliken S , Swindler J. Effects of a remote antimicrobial stewardship program on antimicrobial use in a regional hospital system. Pharmacy (Basel) 2020;8. 10.3390/pharmacy8010041 PMC7151691 32188001

[27] Laible BR , Grosdidier J , Nazir J. Developing an antimicrobial stewardship program across a rural health system: the Avera Health experience. Am J Health Syst Pharm 2019;76:108–113. 31408091 10.1093/ajhp/zxy012

[28] Shively NR , Moffa MA , Paul KT , et al. Impact of a telehealth-based antimicrobial stewardship program in a community hospital health system. Clin Infect Dis 2020;71:539–45. 31504367 10.1093/cid/ciz878

[29] Stenehjem E , Hersh AL , Buckel WR , et al. Impact of implementing antibiotic stewardship programs in 15 small hospitals: a cluster-randomized intervention. Clin Infect Dis 2018;67:525–532. 29790913 10.1093/cid/ciy155

[30] Vento TJ , Veillette JJ , Gelman SS , et al. Implementation of an infectious diseases telehealth consultation and antibiotic stewardship program for 16 small community hospitals. Open Forum Infect Dis 2021;8:ofab168. 34141816 10.1093/ofid/ofab168 PMC8205263

[31] Wilson BM , Banks RE , Crnich CJ , et al. Changes in antibiotic use following implementation of a telehealth stewardship pilot program. Infect Control Hosp Epidemiol 2019;40:810–814. 31172905 10.1017/ice.2019.128

[32] Ceradini J , Tozzi AE , D’Argenio P , et al. Telemedicine as an effective intervention to improve antibiotic appropriateness prescription and to reduce costs in pediatrics. Ital J Pediatr 2017;43:105. 29149862 10.1186/s13052-017-0423-3 PMC5693570

[33] Monkowski D , Rhodes LV , Templer S , et al. A retrospective cohort study to assess the impact of an inpatient infectious disease telemedicine consultation service on hospital and patient outcomes. Clin Infect Dis 2020;70:763–770. 31002338 10.1093/cid/ciz293

[34] Assimacopoulos A , Alam R , Arbo M , et al. A brief retrospective review of medical records comparing outcomes for inpatients treated via telehealth versus in-person protocols: is telehealth equally effective as in-person visits for treating neutropenic fever, bacterial pneumonia, and infected bacterial wounds? Telemed J E-health 2008;14:762–768. 18954245 10.1089/tmj.2007.0128

[35] Tande AJ , Berbari EF , Ramar P , et al. Association of a remotely offered infectious diseases econsult service with improved clinical outcomes. Open Forum Infect Dis 2020;7:ofaa003. 31988969 10.1093/ofid/ofaa003 PMC6976540

[36] Canterino JE , Wang K , Golden M. Provider satisfaction with infectious diseases telemedicine consults for hospitalized patients during the COVID-19 pandemic. *Clin Infect Dis* 2021;ciab479. 10.1093/cid/ciab479 PMC8244553 34048567

[37] Abdel-Massih RC , Mellors JW. Telemedicine and infectious diseases practice: a leap forward or a step back? Open Forum Infect Dis 2019;6:ofz196. 31139674 10.1093/ofid/ofz196 PMC6527083

[38] Suzuki H , Perencevich E , Goto M , et al. A comprehensive assessment of carbapenem use across 90 Veterans’ Health Administration hospitals with defined stewardship strategies for carbapenems. J Antimicrob Chemother 2021;76:1358–1365. 33523128 10.1093/jac/dkab008

[39] Morrill HJ , Gaitanis MM , LaPlante KL. Antimicrobial stewardship program prompts increased and earlier infectious diseases consultation. Antimicrob Resist Infect Control 2014;3:12. 24742249 10.1186/2047-2994-3-12 PMC4018501

[40] Strymish J , Gupte G , Afable MK , et al. Electronic consultations (e-consults): advancing infectious disease care in a large veterans affairs healthcare system. Clin Infect Dis 2017;64:1123–1125. 28158475 10.1093/cid/cix058

[41] Meredith J , Onsrud J , Davidson L , et al. Successful use of telemedicine infectious diseases consultation with an antimicrobial stewardship-led *Staphylococcus aureus* bacteremia care bundle. Open Forum Infect Dis 2021;8:ofab229. 34189171 10.1093/ofid/ofab229 PMC8231364

[42] Gonzalez BE , Sabella C , Esper FP , et al. Physician-to-physician electronic consultation: a tool for the pediatric infectious diseases specialist to document encounters and quantify effort. J Pediatr Infect Dis Soc 2021;10:334–336. 10.1093/jpids/piaa041 32369170

[43] Wood BR , Bender JA , Jackson S , et al. Electronic consults for infectious diseases in a united states multisite academic health system. Open Forum Infect Dis 2020;7:ofaa101. 32328507 10.1093/ofid/ofaa101 PMC7166117

[44] Murthy R , Rose G , Liddy C , Afkham A , Keely E. eConsultations to infectious disease specialists: questions asked and impact on primary care providers’ behavior. Open Forum Infect Dis 2017;4:ofx030. 28470015 10.1093/ofid/ofx030 PMC5407213

[45] Mashru J , Kirlew M , Saginur R , Schreiber YS. Management of infectious diseases in remote northwestern Ontario with telemedicine videoconference consultations. J Telemed Telecare 2017;23:83–87. 26748393 10.1177/1357633X15625136

[46] Rose J , Crosbie M , Stewart A. A qualitative literature review exploring the drivers influencing antibiotic over-prescribing by GPs in primary care and recommendations to reduce unnecessary prescribing. Perspect Public Health 2021;141:19–27. 31633458 10.1177/1757913919879183

[47] Gomez T , Anaya YB , Shih KJ , Tarn DM. A qualitative study of primary care physicians’ experiences with telemedicine during COVID-19. J Am Board Fam Med 2021;34:S61–S70. 33622820 10.3122/jabfm.2021.S1.200517

[48] Suzuki H , Marra AR , Hasegawa S , et al. Outpatient antibiotic-prescribing for common infections via telemedicine versus face-to-face visits: systematic literature review and meta-analysis. *Antimicrob Stewardship Healthcare Epidemiol* 2021;1:e24. 10.1017/ash.2021.179 PMC9495625 36168456

[49] Core elements of outpatient antibiotic stewardship. Centers for Disease Control and Prevention website. https://www.cdc.gov/antibiotic-use/community/pdfs/16_268900-A_CoreElementsOutpatient_508.pdf. Accessed May 18,2021.

[50] Johnson KM , Dumkow LE , Burns KW , Yee MA , Egwuatu NE. Comparison of diagnosis and prescribing practices between virtual visits and office visits for adults diagnosed with sinusitis within a primary care network. Open Forum Infect Dis 2019;6:ofz393. 31660415 10.1093/ofid/ofz393 PMC6778270

[51] Johnson KL , Dumkow LE , Salvati LA , Johnson KM , Yee MA , Egwuatu NE. Comparison of diagnosis and prescribing practices between virtual visits and office visits for adults diagnosed with uncomplicated urinary tract infections within a primary care network. Infect Control Hosp Epidemiol 2021;42:586–591. 33118916 10.1017/ice.2020.1255

[52] Pedrotti CHS , Accorsi TAD , De Amicis Lima K , et al. Antibiotic stewardship in direct-to-consumer telemedicine consultations leads to high adherence to best practice guidelines and a low prescription rate. Int J Infect Dis 2021;105:130–134. 33578013 10.1016/j.ijid.2021.02.020

[53] National survey reveals barriers to outpatient antibiotic stewardship efforts. The PEW Charitable Trusts website. https://www.pewtrusts.org/-/media/assets/2020/08/nationalsurveyrevealsbarriersoutpatientantibioticstewardshipefforts.pdf Accessed on May 23, 2021.

[54] Weiner JP , Bandeian S , Hatef E , Lans D , Liu A , Lemke KW. In-person and telehealth ambulatory contacts and costs in a large US insured cohort before and during the COVID-19 pandemic. JAMA Netw Open 2021;4:e212618. 33755167 10.1001/jamanetworkopen.2021.2618 PMC7988360

